# Quality of occlusal outcome following space closure in cases of lower second premolar aplasia using lingual orthodontic molar mesialization without maxillary counterbalancing extraction

**DOI:** 10.1186/s13005-018-0176-2

**Published:** 2018-09-24

**Authors:** Elisabeth Klang, Frauke Beyling, Michael Knösel, Dirk Wiechmann

**Affiliations:** 1 Private Practice, Lindenstraße 44, Bad Essen, 49152 Germany; 2 Department of Orthodontics, University Medical Center UMG, Robert-Koch-Straße 40, 37075 Göttingen, Germany; 3 Department of Orthodontics, Universidad de La Frontera UFRO, Avenida Francisco Salazar, 01145 Temuco, Chile; 4 Private Practice, Lübecker Straße 128, Hamburg, 22087 Germany; 5 Department of Orthodontics, Hannover Medical School MHH, Carl-Neuberg-Straße 1, 30625 Hannover, Germany

## Abstract

**Background:**

Controlled space closure in cases of isolated lower second premolar aplasia (ILSPA) without maxillary counterbalancing extraction is challenging. Anterior anchorage loss may occur during space closure resulting in compromised occlusal results in terms of an absence of proper canine guidance during laterotrusive mandible movements.

In order to evaluate the effectiveness of Herbst telescope anchorage in combination with double-cable, pull mechanics and a completely customized lingual appliance for orthodontic space management in cases of ILSPA, we tested the null hypothesis that there is a significant deterioration in the sagittal canine relationship towards an Angle-Class-II occlusion expressed as a loss of anterior anchorage following space closure with molar mesialization.

**Methods:**

Twenty-five consecutively de-bonded subjects (female / male 17 / 8; aged at T0 (start of MB Tx) 12.3 to 20.6 years; mean age 15.0 / SD 1.7 years) were included in this retrospective analysis using the inclusion criteria of least of one lower second premolar aplasia; completed treatment with a totally customized lingual appliance (CCLA) in combination with Herbst telescopes. Exclusion criteria were the absence of counterbalancing maxillary extractions, as well as additional tooth aplasia other than lower second premolars. A total of 33 single, lower premolar aplasia space closures (right / left sided 17 / 16) were assessed using plaster casts and intra-oral photographs scaled to the plaster casts, at bonding (T0), Herbst insertion (T1), following gap closure (T2) and de-bonding (T3). Parallelism of roots was controlled by panoramic x-rays at T3.

**Results:**

The mean aplasia space at T0 was 7.5 mm (SD 2.6). Complete space closure was achieved in all 33 situations. The null hypothesis was rejected. There was a significant improvement in the initial canine relationships (mean 3.5 mm distal occlusion at T0) to a mean 0.1 mm at T3. When evaluated against the individual treatment plan, the following amounts of planned improvements were achieved: space closure 100%, canine relationship 97.5%, overjet 93.9%, overbite 96.4%, parallel roots in space closure site 93.9%.

**Conclusion:**

Herbst telescope anchorage in combination with double-cable pull mechanics and a CCLA for orthodontic space closure can deliver predictable, high-quality treatment results.

## Background

Finding adequate solutions for space management and occlusal adjustments in cases of isolated lower second premolar aplasia (ILSPA) is a frequently encountered challenge in clinical orthodontics, implantology and prosthodontics. The types of teeth affected by aplasia are most commonly second premolars, followed by maxillary lateral incisors and maxillary second premolars [1, 2]. In contrast to patients with multiple dental aplasia, patients with ILSPA and otherwise complete dentition, including third molars, may benefit more from orthodontic gap closure than opting for either prosthodontic solutions, auto-transplantation or implants [3, 4].

Third molars, which are often extracted in patients with complete dentition, may be used in this situation to compensate for excess space in the premolar aplasia region. Orthodontic space closure can be performed whilst the patient is a teenager without having to wait for growth to be completed, as required in implantological or prosthodontic treatment approaches [3, 4]. A choice between these treatment options requires that the orthodontist exercises diligence in informing the patient and / or his guardians. For underage patients, decisions are most often made by their guardians and the orthodontist, and it is worth mentioning that the chosen solution for restoring the edentulous space will affect the patient for a lifetime [3].

While it has been widely regarded as favorable to treat cases of ILSPA in combination with maxillary counterbalancing premolar extraction, in order to achieve a proper molar and canine Angle-Class I occlusion in a convenient manner, concepts for ILSPA space management without maxillary counterbalancing extraction have been suggested as an alternative and valid treatment option [5–7]. Particularly in cases where there is no crowding in the upper arch or even with spacing, a counterbalancing extraction may not be considered appropriate. However, as a loss of canine anchorage may occur during space closure, this treatment approach is challenging: compromised occlusal results measured in terms of the absence of proper canine guidance during laterotrusive mandible movements, as well as midline shifts, may be a consequence of inadequate anchorage control and space closure mechanics [3, 8]. Therefore, good clinical practice suggests increasing anchorage during ILSPA space management by making use of inter-maxillary elastics, −telescopes, or - springs, if this is compatible with individual sagittal requirements, such as the presence of an Angle-Class I or II malocclusion [9].

### Study objective

The aim of this study was to evaluate the effectiveness of Herbst telescope anchorage in combination with double-cable pull mechanics and a completely customized lingual appliance (CCLA) for orthodontic space closure in ILSPA. The quality of the occlusal outcome was determined by the deviation of the final canine occlusion from an Angle-Class-I and its consequences for overbite and overjet. The null hypothesis was that there is a significant deterioration towards an Angle-Class II canine relationship following complete space closure using molar mesialization.

## Subjects

### Ethical approval

This retrospective study received full approval from the ethics commission of the Hannover Medical School (MHH; # 7727_BO_K_2018) prior to the commencement of data collection.

### Patient recruitment

All patients who were treated with a CCLA (WIN; DW LingualSystems; Bad Essen, Germany) in one orthodontic center (Bad Essen, Germany) and de-bonded during the observational period from October 1st 2014 to February 28 2018 were consecutively screened for potential eligibility for this retrospective analysis. All treatment plans were approved by the same clinicians (DW, FB) prior to initiation of orthodontic treatment.

### Inclusion and exclusion criteria

All patients meeting the following inclusion criteria were considered as potentially eligible:

(I-1) completion of second dentition including eruption of second molars;

(I-2) unilateral or bilateral aplasia of the lower second premolar;

(I-3) a treatment plan for lower molar mesialization, because of existing third molar;

(I-4) completed treatment with a completely customized lingual appliance (CCLA) in combination with a Herbst appliance.

Exclusion criteria were:

(E-1) additional mandibular tooth aplasia other than lower second premolar or third molar aplasia;

(E-2) maxillary tooth aplasia other than third molar aplasia;

(E-3) counterbalancing maxillary extractions.

To minimise the risk of bias, no patient was excluded from this study for any other reason; in particular, bad compliance or missing records. This procedure was followed in order to evaluate not only the feasibility but, first and foremost, the predictability of the method.

### Patients included in the study

Of all CCLA treatments completed during the recruitment period (de-bonding between October 1st 2014 and February 28 2018), 123 patients (7%) were treated with a combination of a CCLA and a Herbst appliance. Twenty-five subjects with an age range of 12.3 to 20.6 years (mean age 15.0 years/ SD 1.7 years) at the beginning of fixed lingual orthodontic treatment were eligible for trial assessments. A total of 33 situations with lower premolar aplasia space closures (right / left sided 17 / 16) were assessed. Eight patients had a bilateral aplasia, with unilaterally missing second premolars apparent in 17 situations.

### Time points of assessment

Metric assessments were performed at the following time points: T0, immediately prior to bonding of the fixed lingual appliance; T1, initiation of anchorage reinforcement by adding Herbst telescopes; T2, completion of gap closure and removal of Herbst telescopes; T3, de-bonding of the fixed lingual appliance.

The mean ages of the patients at the single treatment steps between T0 and T3 are given in Table 1.

**Table 1:** Descriptive analysis of patients’ ages at selected time points (T0, immediately prior to bonding of the fixed lingual appliance; T1, initiation of anchorage reinforcement by adding Herbst telescopes; T2, completion of space closure and removal of Herbst telescopes; T3, de-bonding of the fixed lingual appliance)

	T0	T1	T2	T3
Age [years]: Mean	15.0	16.0	17.2	18.2
(SD; min.; max.)	(1.7; 12.3; 20.6)	(1.8; 13.3; 21.8)	(1.8; 14.7; 22.9)	(2.0; 15.5; 24.7)

## Methods

### Mechanics used for orthodontic space closure

**Fig. 1:**
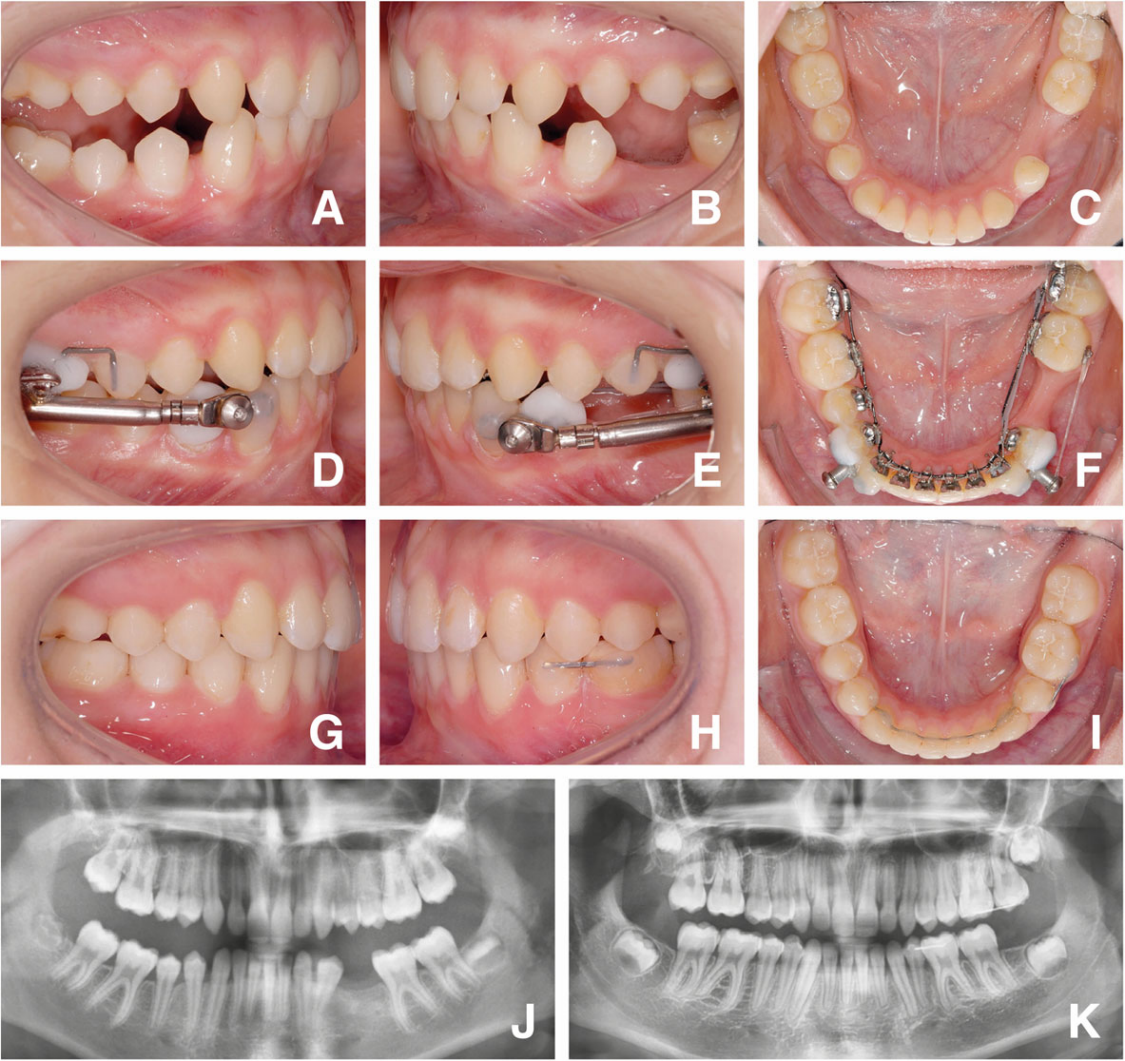
Presentation of a case: Initial situation prior to bonding of the fixed lingual appliance at T0 (**a**, **b**, **c**); Figs. A and B were taken following pre-treatment with removable functional appliances, hence the mandible is seen in a protruded sagittal position. Following leveling and aligning and initiation of anchorage reinforcement by adding Herbst telescopes at T2 (**d**, **e**, **f**), and at T3 following de-bonding of the fixed lingual appliance. Posterior maxillary 6–7 retainers aid in maintaining the vertical relation until lower third molars are brought into occlusal contact, while mandibular 4–6 retainers prevent re-opening of aplasia spaces (**g, h, i**). Panoramic x-rays show an adequate root parallelism after completion of aplasia space closure (**j, k**)

The treatment involved the use of a completely customized lingual appliance (WIN, DW LingualSystems, Bad Essen; Germany) in combination with a Herbst appliance (modified MiniScope, American Orthodontics, USA) for anchorage reinforcement [10]. Second molar brackets were designed with occlusal pads to reduce antagonistic interference. Space closure was achieved by two power chains as a double-cable mechanic device (Morita Energy Chain, Rocky Mountain Orthodontics) attached both lingually from first premolar to second molar and labially from the Herbst attachment to a buccal cleat on the first molar (Fig. 1f). Initial load was set at 150 cN (1.5 N) per power chain, equal to about 300 cN per protraction mechanic. As the alveolar processus in the area of the gap was, in many cases, extremely thin, the goal was to protract especially the first molar in a slightly mesio-rotated position, in order to prevent a gingival recession on its mesial root. Herbst telescope activation was based on individual requirements in a step-wise manner.

### Retention

In addition to the standard retention protocol of placing a fixed 3–3 retainer, a labial fixed retainer was bonded from lower 4–6 at the de-bonding appointment, in order to prevent the aplasia space re-opening, and another one from upper 6–7, in order to prevent elongation of the upper second molar. In cases of pronounced Angle-class II canine relationship at the beginning of lingual treatment, an activator was prescribed for night-time wear, in order to support the retention of the anterior/posterior correction [11].

### Assessment of space closure, canine relationship, overjet and overbite by plaster casts and plaster target set-up models (T0, T3)

Plaster casts routinely prepared at T0 and T3 were utilized to assess space closure, canine relationship, overjet, and overbite. Wax bites taken in the subject’s centric relation were used to correctly position upper and lower plaster casts. The plaster target set-up models were mounted in the articulator. Assessment of overjet and overbite was performed on plaster models using the maximal distances between the upper and lower incisor labial surface or the maximal vertical incisor overlap. The sagittal canine relationship was determined by assigning a value of zero (0) mm to a neutral (Angle-Class I) canine relationship, i. e., the cusp of the lower first premolar is in contact with the upper canine and the upper first premolar. Deviations from neutral sagittal canine occlusion in the posterior direction (i. e., distal or Angle-Class II occlusion) were taken using a sliding caliper (Dentaurum, Münchener Modell, Ispringen, Germany), and defined as positive distances (+ mm); conversely, deviations in the anterior direction (mesial or Angle-Class III occlusion) were defined as negative values (− mm). All metric assessments were performed manually by one operator (EK) using a sliding caliper.

### Assessment of space closure, canine relationship, overjet, and overbite photographs (T1, T2)

Measurements of the gaps between the first premolar and the first molar, as well as the canine and incisor relationships, were recorded and assessed at time points T1 and T2 using digital, high-resolution, intra-oral photographs (D200, with Nikkor 105 mm; Nikon, Tokyo, Japan). Space dimensions were measured on occlusal photographs perpendicular to the occlusal plane using dental mirrors with dimensions 10.5 × 7.5 cm. Photographs used for assessing the canine relationship, as well as the overbite and overjet, were taken directly (perpendicular to the canine’s labial surface, to avoid any potential errors by distortion), using cheek retractors (NOLA, Chicago, IL, USA), without using mirrors.

In order to obtain the true dimensions of the assessed space magnitude, canines, and incisors on the digital photographs, a calibration technique which had been proposed previously was employed [12]. The intraoral photographs were scaled to the corresponding plaster casts by adjusting the dimensions of the upper canine (for sagittal occlusion assessments in lateral view) or the lower first molar (for aplasia space dimension assessments in occlusal-view) and the incisors in the photograph to its corresponding dimensions taken from direct cast measurements. Deviations from neutral sagittal canine occlusion in the posterior direction (i. e., distal or Angle-Class II occlusion) were likewise taken using a sliding caliper.

### Assessment of axial root inclination by panoramic x-rays

Axial root inclination or the absence of parallelism of the roots was evaluated using panoramic x-rays directly following de-bonding (T3). Root angulation of teeth located in a mesial or distal juxtaposition to the site of aplasia (lower first premolars and molars) were judged using the index for root parallelism of the American Board of Orthodontics (ABO; [13]) which distinguishes between roots that are parallel, non-parallel, or contacting.

### Method error analysis

In order to assess the method error for the measurements on plaster casts and intra-oral photographs, ten arbitrarily selected cases were evaluated at T0 (plaster casts) and T1 (please refer to photographs). These measurements were repeated after two weeks:

$$ \mathrm{Me}=\sqrt{\frac{\sum {d}^2}{2n}} $$

where *d* is the difference between the Measurements and *n* the number of assessments [14]. The results of the method error analysis are given in Table 2.

**Table 2:** Error analysis for the two methods used

Method error	Space magnitude	Canine relationship	Overjet	Overbite
T0 [mm] plaster cast	0.19	0.27	0.35	0.16
T1 [mm] photograpy	0.49	0.47	0.30	0.53

### Statistical data analysis

Measurement data were analyzed descriptively (mean, minimum, and maximum values with standard deviations). A comparison of dependent variables was carried out using the t-test.

The significance level was set to α = 5%. SPSS Statistics V_25 software for Windows 10 was used for all statistical tests (IBM, Armonk, NY, USA).

## Results

### Space closure

On completion of fixed lingual orthodontic treatment (T3), all second premolar aplasia spaces were completely closed, as planned in the target set-up, yielding a success rate of 100% (Table 3). With space closure and Angle-class II correction together, the molar relationship on the aplasia side was corrected on average by 10.9 mm.

**Table 3:** Occlusal features: Descriptive analysis of space magnitude and sagittal inter-canine relationships for the aplasia side, overjet and overbite at the selected assessment time points

	T0	T1	T2	T3
Space magnitude [mm]: Mean	7.5	6.5	0.0	0.0
(SD; min.; max.)	(2.6; 2.5; 11.0)	(2.3; 1.5; 11.5)	(0.0; 0.0; 0.0)	(0.0; 0.0; 0.0)
Canine relationship [mm]: Mean	3.5	4.3	0.2	0.1
(SD; min.; max.)	(2.2; 0.0; 7.0)	(2.2; 0.5; 7.7)	(1.0; −1.2; 3.3)	(0.3; 0.0; 1.0)
Overjet [mm]: Mean	4.0	5.1	0.9	1.9
(SD; min.; max.)	(1.6; 2.0; 8.0)	(2.3; 1.1; 10.2)	(0,5; 0.0; 1.8)	(0.4; 1.0; 3.0)
Overbite [mm]: Mean	4.4	3.0	1.0	2.2
(SD; min.; max.)	(1.4; 2.0; 6.5)	(1.2; 0.9; 6.0)	(0.9; −1.1; 3.1)	(0.6; 1.5; 4.0)

Deviations from neutral sagittal canine relationship towards an Angle-Class II were defined as positive distances (+ mm); conversely, deviations in the anterior direction towards an Angle-Class III were defined by negative values (− mm). All values are given in mm

Following leveling and aligning (T1, Table 3), the mean aplasia space dimension was 6.5 mm. Overall duration of space closure produced a mean of 13.0 months (SD 5.5; min. / max. 3.6 / 25.7 months), equal to 0.57 mm per month (SD 0.26; min. / max. 0.11 / 1.35 mm). The differences between right and left sides were minimal (right-sided: 0.59 mm / months; SD 0.26; min. / max. 0.28 / 1.35 mm/ months; left-sided: 0.54 mm / months; SD 0.26; min. / max. 0.11 / 1.25 mm/ months).

### Canine relationship

**Fig. 2:**
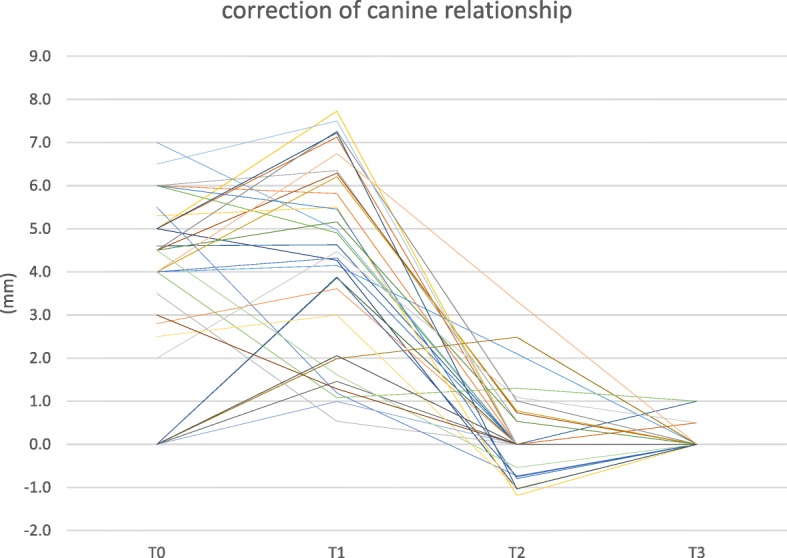
Canine relationship development during treatment at the site of aplasia. The deviation from a canine-Angle Class-I is given in mm. See text for details

**Fig. 3:**
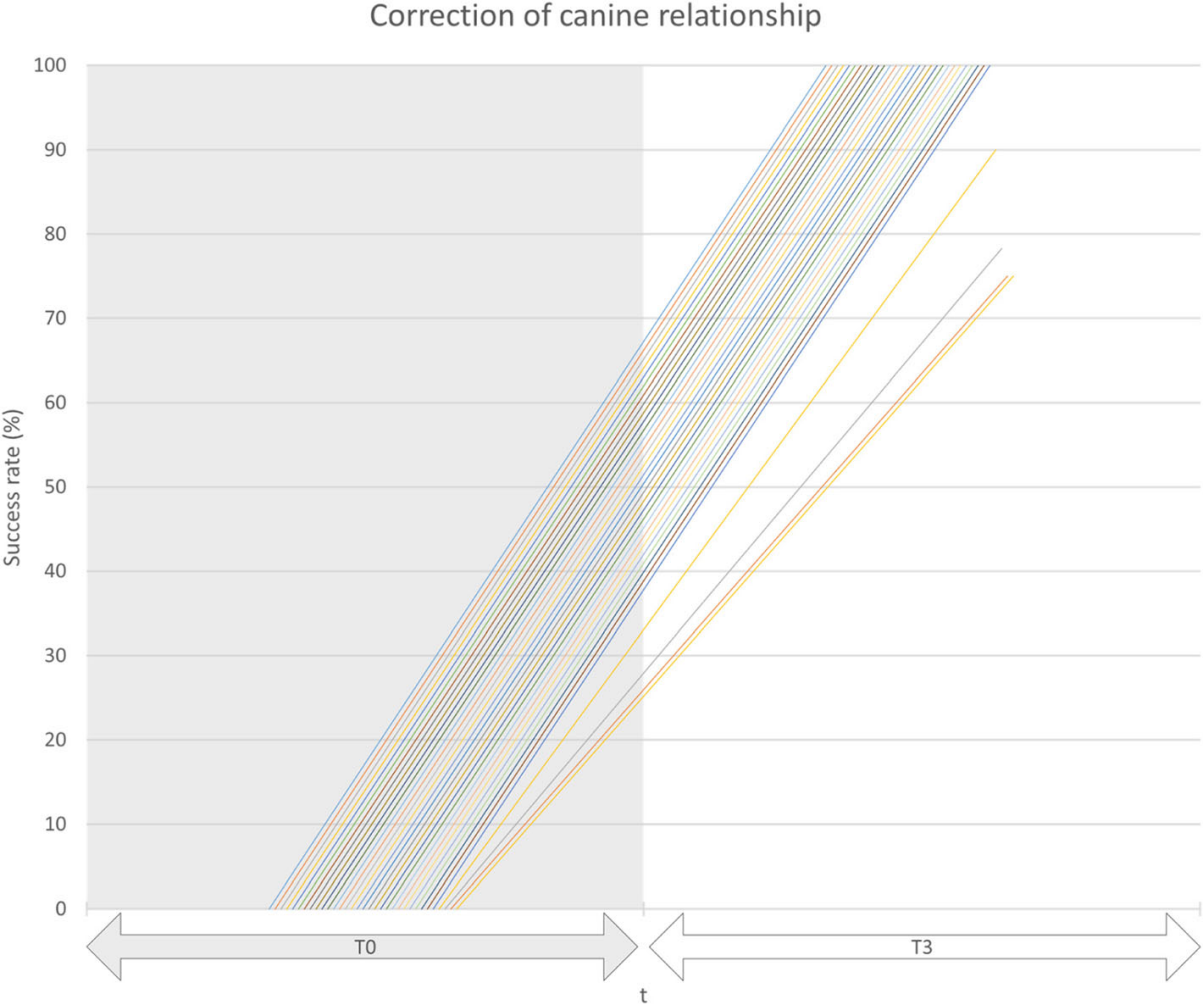
Success rate for canine relationship correction compared to the individual treatment plan (target set-up)

On the aplasia side, at T0 (Table 3) a pronounced tendency for having a distal occlusion was observed, with a mean deviation of 3.5 mm. At T1, i. e., following leveling and aligning, the mean extent of distal occlusion increased insignificantly (*p*-value 0.064) to 4.3 mm, and dropped significantly following Herbst appliance removal (T2) (p-value 0.001) to a mean of 0.2 mm (Fig. 2). In 29 of the 33 canine relationships assessed, an Angle-class I sagittal canine relationship (0 mm) was achieved at T3, with an overall correction of 97.5% compared to the individual treatment plan, the target set-up (Fig. 3). An improvement in distal occlusion compared to T0 was seen in all patients at T2. No significant changes in inter-canine relationships were noted between T2 and T3.

### Overjet and overbite correction

**Fig. 4:**
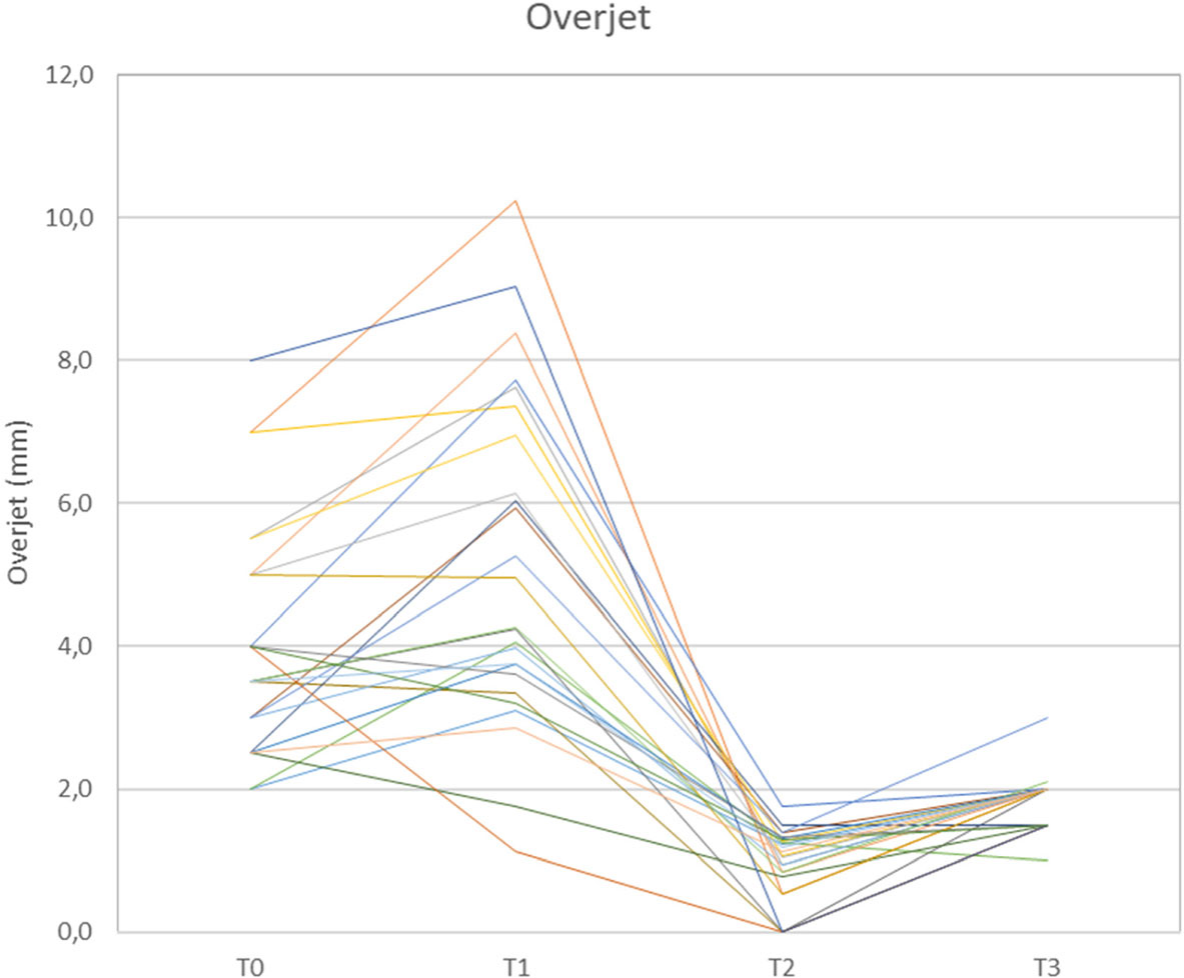
Overjet development (in mm) during treatment. In cases with an initial Angle-Class II-malocclusion, the sagittal incisor relation has been temporarily over-corrected into an edge-to-edge relation at T2

At T0, the mean overjet was 4.0 mm (SD 1.6 mm) and increased highly significantly to a mean 5.1 mm (SD 2.3) following leveling and aligning (T1). It decreased highly significantly to a mean 0.9 mm (SD 0.5) following the Herbst telescope treatment stage, with some sagittal overcorrection (0.9 mm, Table 3, Fig. 4) to address potential relapse in those patients who initially had distal occlusion. At T3, mean overjet was 1.9 mm (SD 0.4 mm). The mean overjet correction was 2.1 mm (93.9% of the planned movements).

**Fig. 5:**
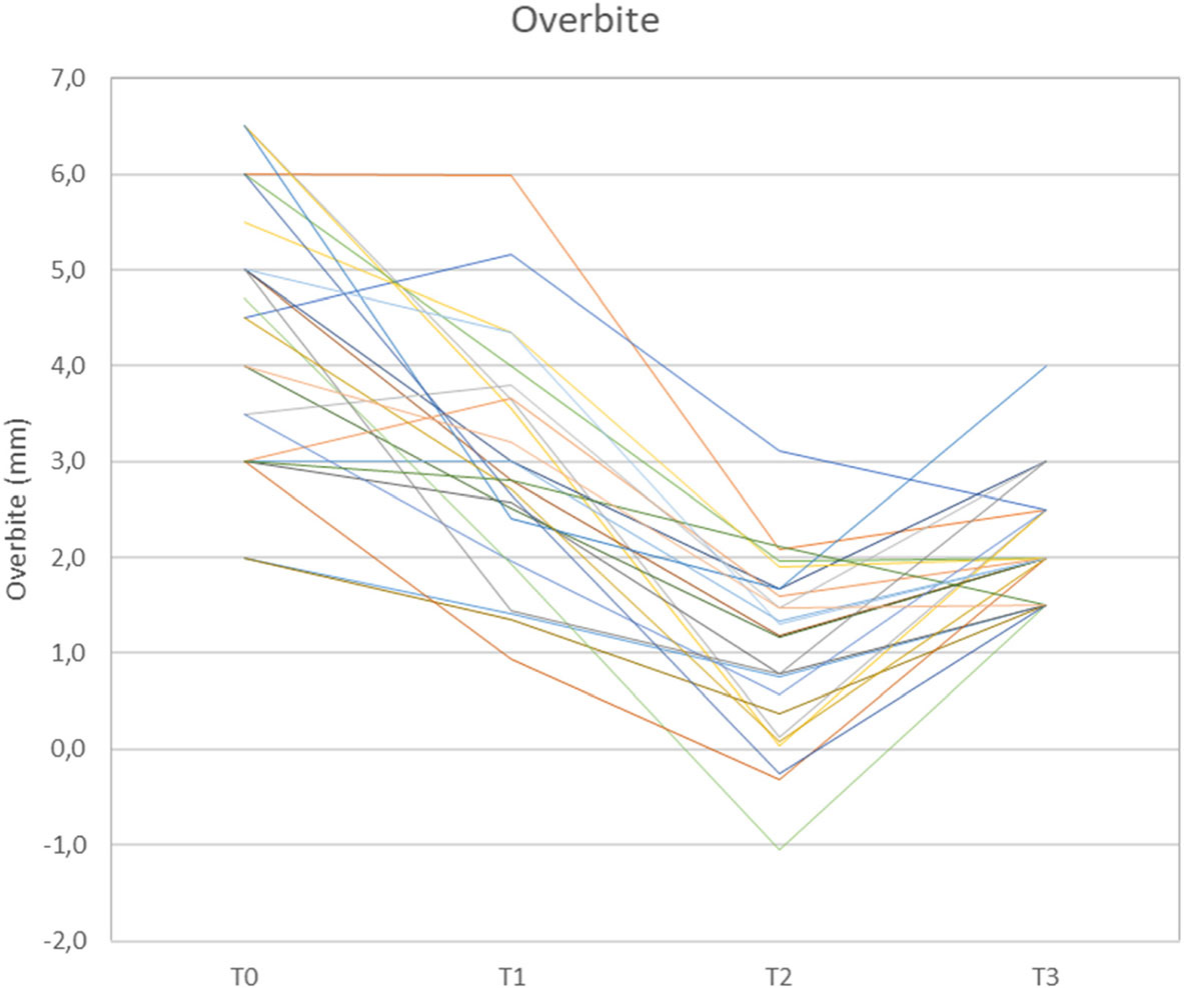
Overbite development (in mm) during treatment

Initial mean overbite was 4.4 mm (SD: 1.4, Table 3, Fig. 5). In all 25 patients, correction of deep bite was achieved by a mean 2.3 mm (SD: 1.2; min. / max. 0.5 / 4.5 mm) representing 96.4% of the planned movements. There was a significant reduction in overbite during leveling and aligning (T1), with an additional reduction during Herbst treatment (T1-T2). Mean overbite at the end of treatment was 2.2 mm (SD 0.6).

### Root angulation following space closure

In 31 of 33 cases (93.9%), the roots of the lower first premolars and those of the first molars were parallel following aplasia space closure. The roots adjacent to two aplasia situations (6.1%) were not parallel at T3. However, contact between adjacent roots was not seen in any of the 33 cases.

### Treatment duration

Total mean duration of treatment with the fixed lingual appliance (T0-T3) was 38.4 months (SD 7.6; min./max. 19.6 months; 52.4 months). Leveling and aligning (T0-T1) took a mean 12.0 months (SD 3.4; min./max. 7.6 / 21.9 months), while Herbst treatment (T1-T2) stage took a mean 13.8 months (SD 4.1; min./max. 3.6 / 21.6 months), with active aplasia space closure durations of 13.0 months. Further occlusal adjustments following space closure (T2-T3) took a mean 12.6 months (SD 5.0; min. / max. 4.6; 22.3 months).

## Discussion

To the best of our knowledge, the present study is probably the first study to provide evidence regarding the rarely addressed subject of molar mesialisation using Herbst appliance anchorage in cases of lower aplasia gap closure without counterbalancing extractions. Clinical recommendations have been based on case reports [3, 8] and retrospective studies on a smaller number of patients, treated for 2.2–3.1 years, with push-and-pull mechanics [5, 7] or Jasper-Jumper [6].

### Study design

This study had a retrospective design. However, all patients who were de-bonded within the time frame of forty-one months prior to starting any assessments were screened for eligibility. In order to evaluate the predictability of the concept, no subject who met the inclusion criteria was excluded because of a lack of compliance, missing records, insufficient oral hygiene, or similar reasons.

### Method error

The reproducibility of the methods used here is judged to be adequate for assessing the occlusal changes in this study, as the variation in repeated plaster cast measurements varied between 0.16 and 0.35 mm, and for the photograph method between 0.3 and 0.53 mm (Table 2).

### Null hypothesis

The null hypothesis that there would be a significant deterioration in the inter-canine relationship towards an Angle-Class-II occlusion, in terms of a loss of anterior anchorage following space closure by molar mesialization, was rejected. There was not only no deterioration in the initial mean sagittal canine relationships (mean 3.5 mm deviation at T0), but instead a highly significant (*p* <  0.001) reduction to a mean 0.1 mm at T3 (Tables 3 and 4).

**Table 4:** Space magnitude, sagittal inter-canine relationships, overjet, and overbite: Non-parametric comparison of dependent samples using the t-test

p-Value	T0 – T1	T0 – T2	T0 – T3	T1 – T2	T1 - T3	T2 – T3
Space magnitude	0.01	< 0.001	< 0.001	< 0.001	< 0.001	n. s.
Canine relationship	n. s.	< 0.001	< 0.001	< 0.001	< 0.001	n. s.
Overjet	0.002	< 0.001	< 0.001	< 0.001	< 0.001	< 0.001
Overbite	< 0.001	< 0.001	< 0.001	< 0.001	0.003	< 0.001

Difficulties in achieving proper canine guidance in cases of ILSPA – mainly due to biomechanical requirements - have been described previously [3, 6]. Pure mesialization is desired in cases of ILSPA in combination with Angle-Class I occlusion, but reciprocal effects of common space closure mechanics contribute to a typical loss of anchorage and resulting midline shifts [8]. Moreover, an Angle-Class II occlusion in combination with ILSPA elevates the treatment difficulty level clearly. Therefore, Herbst appliance or Jasper-Jumper anchorage has been proposed to limit the reciprocal effects of space closure on sagittal occlusion [6, 12]. This appears to be advantageous compared to mini-screw anchorage, not only in terms of space closure durations, but particularly in cases in which there is an existing Angle-class II occlusion [12].

### Speed of space closure

The mean speed of space closure was found to be 0.57 mm / month, which is slightly (12%) increased compared to those reported by Metzner et al. (0.51 mm / month) [12]. This may be explained by this study having utilized a Herbst telescope system (WIN appliance, DW Lingualsystems, Bad Essen, Germany) which is less susceptible to appliance fractures or failures [10] than the appliance of the type used by Metzner et al. (Incognito appliance, 3 M Top Service für Lingualtechnik, Bad Essen, Germany) [12, 15]: When using a Herbst appliance or a Jasper-Jumper as anchorage during molar mesialisation, it is crucial to keep the rate of appliance fractures low, in order to achieve reduced treatment duration by a continuous space closure strictly from a posterior direction, without anchorage or interruption of fixed treatment. The Herbst appliance in combination with the CCLA used here is considered to be suitable for this purpose, based on previous research outcomes [10]. The telescopes of the Herbst appliance are not directly connected to the lingual appliance, thereby avoiding arch-wire fractures which would otherwise produce additional repair appointments.

### Overjet and overbite correction

Successful overjet reduction with a satisfying sagittal inter-incisal relation was accomplished in 24 of 25 patients. Reductions in overjet by Herbst therapy has been reported to vary from 3.1 to 6.9 mm. However, the purpose of these studies was to achieve sagittal mandibular enhancement and they were based on samples with larger initial overjets [16–18]. In our study, the Herbst telescopes were used primarily for anchorage reinforcement and, because of the inclusion of patients with initially neutral occlusion, the sample taken may have had less pronounced overjets compared to studies on the subject of Angle-Class II-correction effectiveness.

Deep overbite has been reported to be commonly associated with aplasia [19, 20]. Deep overbite corrections in this study were observed to have a mean of 2.3 mm (min. / max. 0.5 / 4.5 mm; SD 1.2), which is in line with previous reports on Herbst appliance therapy [21, 22]. With the exception of two patients who had a normal overbite by the start of treatment, overbite reduction was finally achieved in all 23 other patients. At T3, three patients had a slightly increased overbite, as a trend, with overbites of 3 or 4 mm, respectively. Although the initial overbite of the latter was, with a value of 6.5 mm, distinctively more pronounced compared to the mean initial overbite of the sample (Table 3), inadequate deep bite correction may be attributed to a lack of proper upper and lower incisor third order control and underlines the need for choosing arch-wires with adequate dimensions or third order overcorrections in cases of ILSPA.

### Root angulation following space closure

Parallelism of the roots of the first molar and those of the first premolar following gap closure was observed in 31 out of 33 situations. In two situations a mesial angulation of the premolar roots was observed and this is most likely due to using elastic chains with an under-dimensioned, incompletely slot filling 0.016 × 0.024 “steel arch-wire (slot dimensions: 0.018 x 0.025”), with some proportion of a vertical bowing effect. Bracket slots with larger mesio-distal dimensions may reduce this problem.

### Treatment duration and clinician’s experience

**Fig. 6:**
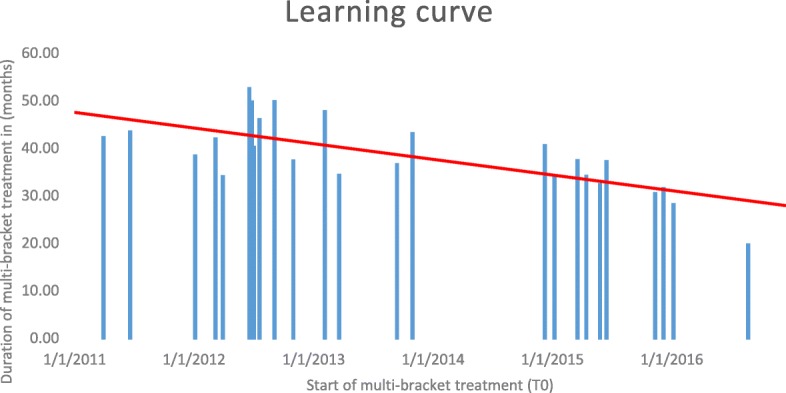
Treatment duration related to start of CCLA treatment: The learning curve as a trend line is indicated in red

Despite the existence of several reports on the orthodontic treatment of ILSPA, information about treatment duration is scarce [5–7]. Zimmer and Rottwinkel [6] reported a duration of mean active ILSPA space closure treatment of 3.1 years (37 months)(min. / max. 2.4 / 3.1 months), which is similar to our results, although the extent of molar mesialisation during orthodontic space closure reported by those authors was smaller than in our patients. Orthodontic treatment duration in those cases of ILSPA assessed by this study had a mean of 39.3 months, thereby distinctly exceeding average treatment durations of conventionally / labially bonded orthodontic cases without the need for space closure by congenitally missing teeth (20.02 mo; [23]). Levelling and aligning required a mean 12.0 months in the actual study cases. However, we observed a decrease in average treatment duration with increasing experience collected while treating the first patients of this study. The experience of the clinician seems to have an influence and Fig. 6 provides some evidence of the effect of an increasing training curve, reflected in a manifest reduction in mean treatment times of patients bonded later during the observation time frame. Based on the experiences gained whilst treating these patients, it is advisable to use elastic chains between lower first premolars early in treatment, in order to reduce the time until incorporation of the Herbst telescopes. Also, the use of steel ligatures early in treatment is considered to be helpful in angulation control of lower canines and may indeed speed up treatment.

### Alternatives to aplasia space closure

ILSPA gap management can be addressed by a variety of diverse treatment approaches, each of which having both advantages and disadvantages [3]. Potential treatment options mostly involve abundant mesio-distal enamel reduction of deciduous second molars, in order to prepare these teeth for a premolar-shaped crown. The disadvantages of this approach may be the risk of damaging the pulp during reduction and the questionable long-term prognosis for milk molars with distinctively shorter roots [3, 24]. On the other hand, this approach provides a much quicker solution than orthodontic space closure. Other viable treatment options include the use of bridges, implants, or auto-transplants [3, 25]. A success rate of auto-transplanted third molars to replace premolars has been reported to be 85% after 3.3 years, but also requires subsequent orthodontic treatment to prevent increased failure rates due to persistent infra-occlusion and ankylosis [25, 26], which are also the typical problems maintained primary molars [3]. The advantage of protracting molars into edentolous areas would be a much better long-term prognosis and, in the case of the presence of third molars, the opportunity to close the gap without using material that may require renewal at some point in the future. One should bear in mind that ILSPA patients presenting to orthodontists are mostly under-aged and unable to fully evaluate the consequences of the various treatment options. The method proposed by this study offers a sustainable solution for replacing deciduous molars without utilizing extraneous material whilst preserving third molars [5], and also provides for adequate control of typical side-effects, such as anchorage loss or incomplete space closure.

## Conclusion

Based on the results of this retrospective study on ILSPA gap management using Herbst anchorage reinforcement and a double-cable pull mechanic with a completely customized lingual appliance, the following conclusions can be drawn:

- Space closure was achieved in all cases.
- Loss of anchorage during space closure is clinically well controllable, as Herbst anchorage seems to compensate distalizing forces of the double-cable mechanic, thereby providing the basis for adjusting an adequate inter-canine relationship in accordance with the Angle-Class-I occlusion concept.
- The proposed technique is therefore considered to be a clinically viable treatment option for ILSPA gap management.
- The experience level of the clinician appears to have an influence on treatment duration.

## References

[1] Khalaf K, Miskelly J, Voge E, Macfarlane TV (2014). Prevalence of hypodontia and associated factors: a systematic review and meta-analysis. J Orthod.

[2] Rakhshan V, Rakhshan A (2016). Systematic review and meta-analysis of congenitally missing permanent dentition: sex dimorphism, occurrence patterns, associated factors and biasing factors. Int Orthod.

[3] Kokich VG, Kokich VO (2006). Congenitally missing mandibular second premolars: clinical options. Am J Orthod Dentofac Orthop.

[4] Jung RE, Pjetursson BE, Glauser R, Zembic A, Zwahlen M, Lang NP (2008). A systematic review of the 5-year survival and complication rates of implant-supported single crowns. Clin Oral Implants Res.

[5] Zimmer B, Guitard Y (2001). Orthodontic space closure without contralateral extraction through mesial movement of lower molars in patients with aplastic lower second premolars. J Orofac Orthop.

[6] Zimmer B, Rottwinkel Y (2002). Orthodontic space closure without counterbalancing extractions in patients with bilateral aplasia of the lower second premolars. J Orofac Orthop.

[7] Zimmer B, Schelper I, Seifi-Shirvandeh N (2007). Localized orthodontic space closure for unilateral aplasia of lower second premolars. Eur J Orthod.

[8] Fines C, Rebellato J, Saiar M (2003). Congenitally missing mandibular second premolar: treatment outcome with orthodontic space closure. Am J Orthod Dentofac Orthop.

[9] Fiorentino G, Melsen B (1996). Asymmetric mandibular space closure. J Clin Orthod.

[10] Wiechmann D, Vu J, Schwestka-Polly R, Helms HJ, Knösel M (2015). Clinical complications during treatment with a modified Herbst appliance in combination with a lingual appliance. Head Face Med.

[11] Pancherz H, Ruf S (2008). The Herbst appliance - research-based clinical management.

[12] Metzner R, Schwestka-Polly R, Helms HJ, Wiechmann D (2015). Comparison of anchorage reinforcement with temporary anchorage devices or a Herbst appliance during lingual orthodontic protraction of mandibular molars without maxillary counterbalance extraction. Head Face Med.

[13] Peck JL, Sameshima GT, Miller A, Worth P, Hatcher DC (2007). Mesiodistal root angulation using panoramic and cone beam CT. Angle Orthod.

[14] Dahlberg G (1940). Statistical methods for medical and biological students.

[15] O’Keeffe C (2013). Complications associated with the use of a Herbst appliance in combination with a completely customized lingual appliance. Master-Thesis.

[16] Pancherz H, Hansen K (1986). Occlusal changes during and after Herbst treatment: a cephalometric investigation. Eur J Orthod.

[17] Obijou C, Pancherz H (1997). Herbst appliance treatment of class II, division 2 malocclusions. Am J Orthod Dentofac Orthop.

[18] Bock NC, Ruf S, Wiechmann D, Jilek T (2016). Herbst plus lingual versus Herbst plus labial: a comparison of occlusal outcome and gingival health. Eur J Orthod.

[19] Dermaut L, Goeffers KR, De Smit AA (1986). Prevalence of tooth agenesis correlated with jaw relationship and dental crowding. Am J Orthod Dentofac Orthop.

[20] Carter NE, Gillgrass TJ, Hobson RS, Jepson N, Eechan JG, Nohl FS, Nunn JH (2003). The interdisciplinary management of hypodontia: orthodontics. Br Dent J.

[21] Pancherz H (1982). The mechanism of class II correction in Herbst appliance treatment. A cephalometric investigation. Am J Orthod.

[22] Nelson B, Hägg U, Hansen K, Bendeus M (2007). A long-term follow-up study of class II malocclusion correction after treatment with class II elastics or fixed functional appliances. Am J Orthod Dentofac Orthop.

[23] Tsichlaki A, Chin SY, Pandis N, Fleming PS (2016). How long does treatment with fixed orthodontic appliances last? A systematic review. Am J Orthod Dentofac Orthop.

[24] Bjerklin K, Bennett J (2000). The long-term survival of lower second primary molars in subjects with agenesis of the premolars. Eur J Orthod.

[25] Bauss O, Sadat-Khonsari R, Engelke W, Kahl-Nieke B (2002). Results of transplanting developing third molars as part of orthodontic space management. Part 1: clinical and radiographic results. J Orofac Orthop.

[26] Bauss O, Sadat-Khonsari R, Engelke W, Kahl-Nieke B (2003). Results of transplanting developing third molars as part of orthodontics space management. Part 2: results following the orthodontic treatment of transplanted developing third molars in cases of aplasia and premature loss of teeth with atrophy of the alveolar process. J Orofac Orthop.

